# Magnesium isoglycyrrhizinate attenuates acute alcohol-induced hepatic steatosis in a zebrafish model by regulating lipid metabolism and ER stress

**DOI:** 10.1186/s12986-022-00655-7

**Published:** 2022-03-24

**Authors:** Wencong Dai, Kunyuan Wang, Xinchun Zhen, Zhibin Huang, Li Liu

**Affiliations:** 1 State Key Laboratory of Organ Failure Research, Guangdong Provincial Key Laboratory of Viral Hepatitis Research, Department of Infectious Diseases, Nanfang Hospital, Southern Medical University, Guangzhou, 510515 Guangdong China; 2 Department of Gastroenterology, The Second Affiliated Hospital of Guangzhou Medical University, Guangzhou, 510260 Guangdong China; 3 Department of Infectious Diseases, The Fifth Affiliated Hospital of Sun Yat‐Sen University, Zhuhai, 519000 Guangdong China; 4 Division of Cell, Developmental and Integrative Biology, School of Medicine, South China University of Technology, Guangzhou, 510006 Guangdong China

**Keywords:** Zebrafish, Magnesium isoglycyrrhizinate, Hepatic steatosis, Alcoholic liver disease

## Abstract

**Background:**

Alcoholism is a well-known risk factor for liver injury and is one of the major causes of hepatic steatosis worldwide. Although many drugs have been reported to have protective effects against acute alcohol-induced hepatotoxicity, there is limited available treatment for alcoholic liver disease (ALD), indicating an urgent need for effective therapeutic options. Herein, we first reported the protective effects of magnesium isoglycyrrhizinate (MgIG) on acute alcohol-induced hepatic steatosis and its related mechanisms in a zebrafish model.

**Methods:**

Alcohol was administered directly to embryo medium at 5 days post-fertilization (dpf) for up to 32 h. MgIG was given to the larvae 2 h before the administration of alcohol and then cotreated with alcohol starting at 5 dpf. Oil red O staining was used to determine the incidence of steatosis, and pathological features of the liver were assessed by hematoxylin–eosin staining. Biological indexes, total cholesterol (TC) and triacylglycerol (TG) were detected in the livers of zebrafish larvae. Morphological changes in the livers of zebrafish larvae were observed using liver-specific EGFP transgenic zebrafish (Tg(*lfabp10a:eGFP*)). The expression levels of critical molecules related to endoplasmic reticulum (ER) stress and lipid metabolism were detected by qRT–PCR, whole-mount in situ hybridization and western blotting.

**Results:**

Alcohol-treated larvae developed hepatomegaly and steatosis after 32 h of exposure. We found that MgIG improved hepatomegaly and reduced the incidence of steatosis in a dose-dependent manner by oil red O staining and diminished deposits of alcohol-induced fat droplets by histologic analysis. Moreover, MgIG significantly decreased the levels of TC and TG in the livers of zebrafish larvae. Furthermore, the expression levels of critical genes involved in ER stress (*atf6*, *irela*, *bip*, *chop*) and the key enzymes regulating lipid metabolism (*acc1, fasn, hmgcs1* and *hmgcra*) were significantly higher in the alcohol-treated group than in the control group. However, in the MgIG plus alcohol-treated group, the expression of these genes was markedly decreased compared with that in the alcohol-treated group. Whole-mount in situ hybridization and western blotting also showed that MgIG had an effect on the expression levels of critical genes and proteins involved in lipid metabolism and ER stress. Our results revealed that MgIG could markedly regulate these genes and protect the liver from ER stress and lipid metabolism disorders.

**Conclusions:**

Our study is the first to demonstrate that MgIG could protect the liver from acute alcohol stimulation by ameliorating the disorder of lipid metabolism and regulating ER stress in zebrafish larvae.

**Supplementary Information:**

The online version contains supplementary material available at 10.1186/s12986-022-00655-7.

## Background

Alcoholic liver disease (ALD) is the major cause of liver-related mortality and has become a public health issue worldwide [1, 2]. Humans drink acutely or chronically, and different drinking patterns are considered to markedly affect ALD. Acute alcoholism is now much more common than chronic alcoholism and can cause mitochondrial dysfunction [3], secretory pathway stress [4], ER stressand lipid accumulation in hepatocytes [5]. ER stress refers to the constant unfolded protein response (UPR) activation due to unmitigated accumulation of unfolded proteins in the ER, reflecting organelle dysfunction that can result in hepatic steatosis and cell apoptosis [6]. Previous studies reported that ER stress induced by alcohol and tunicamycin is sufficient to cause hepatic steatosis [7–9]. On the other hand, improving ER stress can reduce ALD [10]. These results point to a role for ER stress as a causative factor for ALD.

Lipid accumulation and metabolic imbalances are among mechanisms of the pathogenesis of ALD. Alcohol-induced liver fatty acid uptake, impairment of fatty acid oxidation, promotion of de novo lipid synthesis, neutral lipid storage and inhibition of lipid export are all pathways leading to lipid accumulation in hepatocytes [11, 12]. Excess lipid deposits in the liver would make hepatocytes more responsive to liver toxins and incur advanced liver injury [13, 14].

Although a considerable amount of attention has been devoted to acute ALD, the underlying mechanisms are not fully clarified, and therapeutic drugs are currently lacking. The identification of molecular targets affected by acute ALD has the potential for developing newer therapeutic strategies. The fact that there is an alarming rise in binge drinking makes this issue even more compelling to address.

Zebrafish models of acute ALD have been well established in recent years and are useful for dissecting the molecular pathogenesis and assessing effective chemical agents for acute ALD [15–19]. In comparison with traditional rodent model organisms, zebrafish have many advantages, including their simple raising system, relatively small body size, fast generation time, and low feeding costs [20]. In a review, Howarth et al. [21] indicated that zebrafish possessed key enzymes to utilize and excrete alcohol, which were functionally analogous to the mammalian system. Moreover, the livers of zebrafish were matured by 4 dpf and could be visualized directly in the transparent body. Importantly, alcohol and other chemical candidates could be simply added to fish water, providing a rapid and effective means to study ALD. Tsedensodnom et al. [6] took advantage of a series of well-established pharmacological inhibitors, such as the ADH1 inhibitor 4-methylpyrazole (4MP), CYP2E1 inhibitor chlormethiazole (CMZ) and antioxidant *N*-acetylcysteine (NAC), and demonstrated that these agents could significantly improve acute alcohol-induced hepatic steatosis in a zebrafish model. Given that the zebrafish model was suitable for studying acute ALD and identifying effective drugs via a relatively simple protocol in vivo, we utilized this model for further study.

Magnesium isoglycyrrhizinate (MgIG) is a magnesium salt of 18-α glycyrrhizic acid stereoisomer and has been commonly used as a hepatoprotective agent to treat chronic hepatitis patients in Asia for many years [22]. MgIG has been shown to possess various beneficial pharmacological effects, including antitumor [23], antiviral [24], anti-inflammatory [25] and hepatoprotective activities [26, 27]. However, its role in acute alcohol-induced hepatic steatosis is rarely reported, and its related mechanisms are unclear.

In the present study, we evaluated the protective effects of MgIG against acute alcohol-induced hepatic steatosis in a zebrafish model. Our results showed that MgIG effectively attenuated hepatomegaly and hepatic lipid accumulation induced by alcohol exposure. MgIG exerted its hepatoprotective effect probably through regulating ER stress and ameliorating the disorder of lipid metabolism in zebrafish larvae.

## Methods

### Zebrafish care and feeding

Wild-type AB strain zebrafish and liver-specific EGFP transgenic zebrafish (Tg(*lfabp10a:eGFP*)) were obtained from the key laboratory of zebrafish modeling and drug screening for human diseases institute, Southern Medical University, Guangzhou, China. Zebrafish were maintained on 14:10 h light:dark cycle at 28.5 °C according to standard procedures (Westerfield, 1995). Fertilized embryos collected following natural spawning were cultured in embryo medium containing methylene blue. This study was approved by the Animal Care and Use Committee of Southern Medical University, Guangzhou and every effort was made to minimize suffering.

### Pharmacological treatments

Embryos were reared in embryo medium to 5 dpf, larvae with normal development were cultured in six well plates and treated with 350 mM alcohol for up to 32 h. Parafilm were used to prevent alcohol volatilization. 0.1 mg ml^−1^ MgIG, 0.05 mg ml^−1^ MgIG and 0.01 mg ml^−1^ MgIG (purity 99.3%, Chia-tai Tianqing Pharmaceutical Co., Ltd, Nanjing, China) were given to the larvae 2 h before the administration of alcohol and then were co-treated with alcohol starting at 5 dpf. *N*-Acetylcysteine (NAC, 20 µM, Sigma-Aldrich, St Louis, USA) as a positive control was given to the larvae 2 h before the administration of alcohol and then were co-treated with alcohol starting at 5 dpf. Tunicamycin (1 µM, Targetmol, Shanghai, China) as a ER stress inducer was given to the larvae at 5 dpf. NAC and tunicamycin dose selection depends on the previous study [6, 28].

### Whole-mount oil red O staining

At the end of the experiment, whole larvae were fixed in 4% paraformaldehyde (PFA) overnight at 4 °C and washed twice with phosphate-buffered saline (PBS), infiltrated with 40%, 60%, 80% and 100% 1,2 propylene glycol 20 min respectively and stained with fresh 0.5% oil red O solution (Cat. No.O0625, Sigma-Aldrich, St. Louis, USA) in 100% 1,2 propylene glycol in the dark overnight at room temperature. Stained larvae were washed with PBS and faded the background color by washing with decreasing concentrations of 1,2 propylene glycol. Larvae were defined positive for hepatic steatosis if the boundary between the liver and surrounding tissue is clear, and three or more lipid droplets were visible within the liver by whole-mount oil red O staining [19].

### Oil red O staining of cryosections

The experiments protocols for details were as previously descried [29].

### Histologic analysis

After sacrificing the larvae, larvae were harvested and washed with phosphate-buffered saline (PBS), then fixed with 4% PFA overnight, embedded in paraffin following the standard procedures. Sections of 4 µm thickness were cut, deparaffinized, hydrated and stained with hematoxylin & eosin (H&E) and observed on BX51 microscope (Olympus, Tokyo, Japan).

### Biological analysis

The livers of 50–60 zebrafish larvae were homogenized in lysis buffer, centrifuged and got supernatant liquid. TC and TG in the liver homogenates were measured using the kits (APPLYGEN Bioengineering, Beijing, China and Nanjing Jiancheng Bioengineering, Nanjing city, China) following the manufacturer’s instructions, and were normalized to the total protein concentration as determined by Bradford Assay (Bio-Rad, Hercules, CA).

### Quantitative RT-PCR

Total RNA was extracted and purified from the livers of zebrafish larvae (n ≥ 30 each group) using RNeasy plus mini kit (Qiagen, German) and generate cDNA using PrimeScript™ RT-PCR Kit with gDNA Eraser (Takara Biotechnology Co.Ltd, Dalian, China). Quantitative reverse-transcription PCR (qRT-PCR) was carried out on Light Cycler 480 (Roche, Basel, Switzerland) with one cycle of 95 °C for 5 min, followed by 45 cycles of 95 °C for 10 s, 60 °C for 20 s and 72 °C for 30 s using gene-specific primers list in Additional file 1: Table S1. Gene was used as a reference, and expression was calculated using the cycle threshold (Ct) method. *eef1a1* was used as a reference, and expression was calculated using the cycle threshold (Ct) method (2^−Ct (target)^/2^−Ct (*eef1a1*)^).

### In vitro synthesis of antisense RNA probes and whole-mount in situ hybridization

Antisense RNA Probes of *bip* and *hmgcs1* were generated by PCR amplification from cDNA generated from RNA of zebrafish larvae. Each fragment was cloned into PBSK and sequenced. Probes were created using digoxigenin RNA Labeling Mix (Roche, Basel, Switzerland) by T7 RNA Polymerase (Roche) using in vitro transcription systems according to a standard protocol. Whole-mount in situ hybridization was performed as described [30]. Stained larvae were imaged with a stereo fluorescence microscope (Olympus Corporation).

### Western blotting

Protein was extracted from a pool of zebrafish livers (n ≥ 50 each group) were lysed in RIPA buffer supplemented with protease inhibitors (Roche). The entire extracts were resolved by SDS-PAGE, transferred to a PVDF membrane (Bio-Rad), blocked with 5% bovine serum albumin (BSA) for 1 h at room temperature and incubated with primary antibodies hmgcs1 (1:1000, Abcam), bip (1:1000, Bioworld), chop (1:1000, Bioworld), perk (1:1000, Abcam), β-actin (1:1000, Abcam) at 4 °C overnight. After the incubation with the corresponding secondary antibodies conjugated to horseradish peroxidase, the signals of the membranes were detected by enhanced chemiluminescence western blotting substrate (Pierce, Rockford).

### Statistical analyses

The results are expressed as mean ± SD. One-way ANOVA test and Chi-Square test are performed for analysis. Graphpad Prism software (Graphpad, La Jolla, CA, USA) was used to draw figures. Differences were considered significantly at *P* < 0.05 level.

## Results

### MgIG effectively attenuated acute alcohol-induced hepatic steatosis in zebrafish larvae

**Fig. 1:**
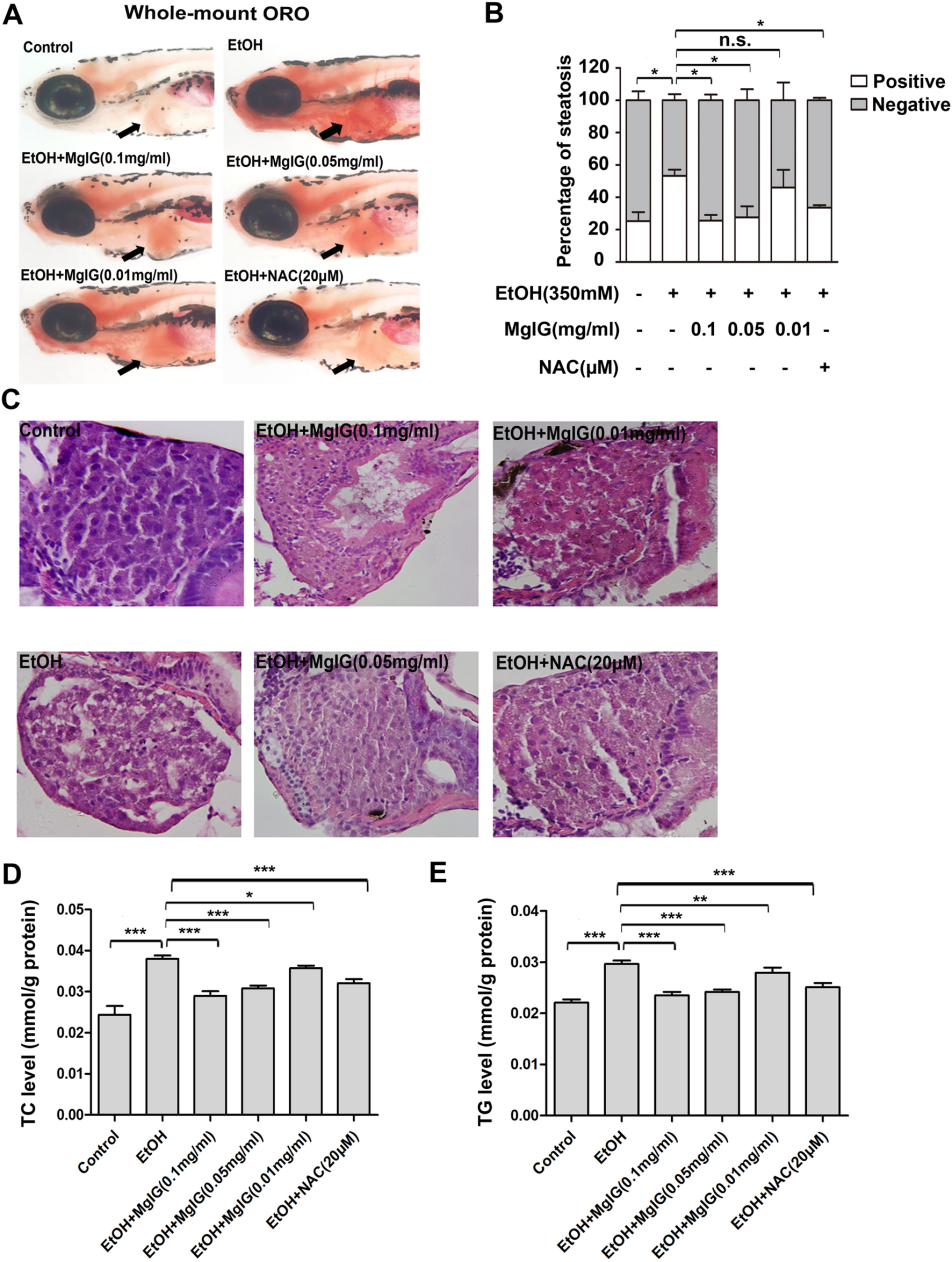
MgIG ameliorate acute alcohol induced-hepatic steatosis in zebrafish Larvae. **a** 5 dpf larvae were treated as indicated for 32 h and stained with whole-mount oil red O. The typical pictures of each group were shown. Arrows point to the liver. **b** Hepatic steatosis was defined as three or more lipid droplets deposition in liver. Bar chart indicates the percentage of larvae with steatosis (n = 97–223 each group; *P < 0.05 by one-way ANOVA)*.* **c** H&E staining of paraffin sections through the livers of larvae treated as indicated. Clear cytoplasmic lipid droplets were seen in livers from larvae fed with alcohol. MgIG treatment decreased lipid droplets and ameliorated hepatic steatosis in alcohol-treated zebrafish larvae**. d** TC levels in the livers of zerafish larvae in each group. **e** TG levels in the livers of zerafish larvae in each group. (n = 50–60 each group; *P < 0.05, **P < 0.01, ***P < 0.001, by one-way ANOVA)

**Fig. 2:**
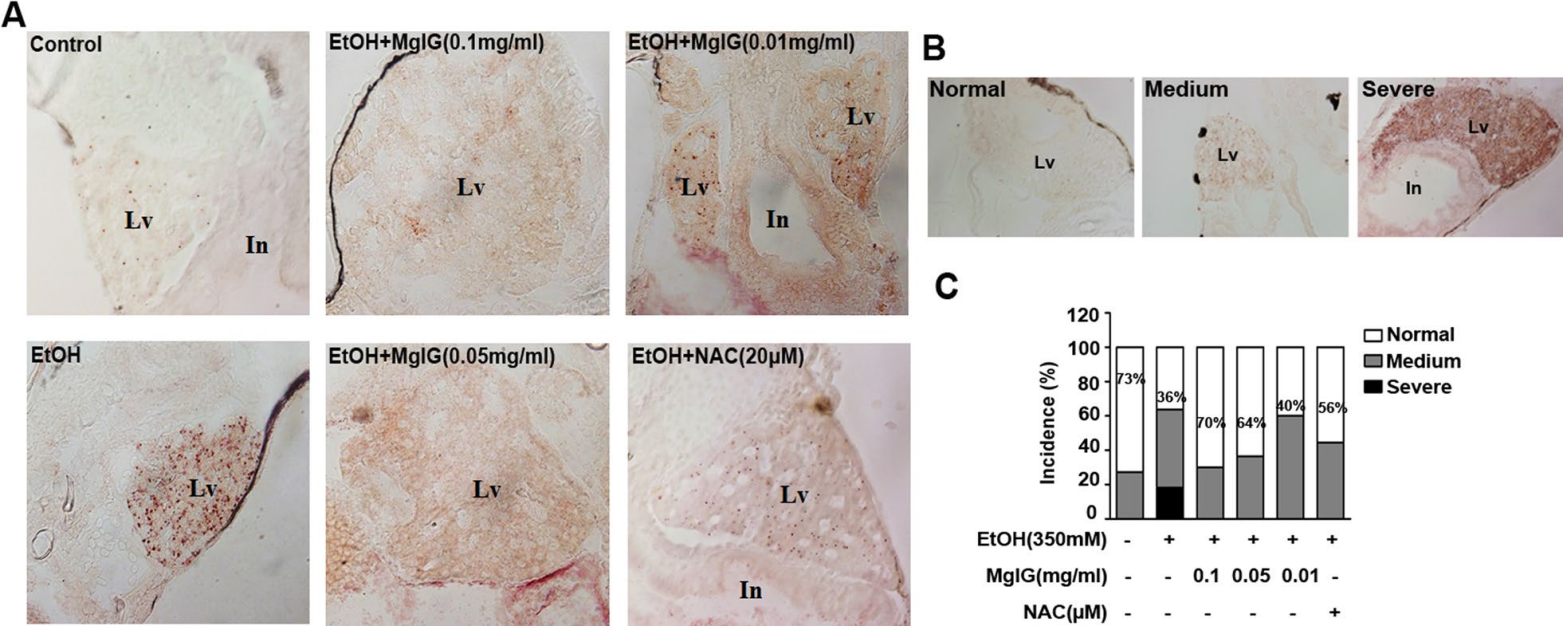
MgIG ameliorate acute alcohol induced-hepatic steatosis in zebrafish Larvae. **a** Oil red O staining of frozen sections through the livers of larvae treated as indicated. Clear lipid droplets were seen in livers from larvae fed with alcohol. MgIG and NAC treatment decreased lipid droplets and ameliorated hepatic steatosis in alcohol-treated zebrafish larvae. **b** Oil red O staining of frozen sections through the livers of zebrafish larvae. We categorized “Normal”, “Medium”, or “Severe” by varying degrees of lipid deposition in the livers of zebrafish by frozen liver sections. Representative images of “Normal”, “Medium”, or “Severe” were shown (× 400 magnification). **c** Quantification of steatosis is categorized “normal”, “medium”, or “severe”. Bar chart indicates the percentages of each category in each group, and the percentages of larvae in “normal” category is noted (n = 9–11 each group)

Various experimental and clinical studies demonstrated that MgIG had protective effects on liver diseases. We investigated whether MgIG could attenuate alcohol-induced hepatic steatosis in zebrafish. First, we observed that acute alcohol exposure induced excessive lipid accumulation in the livers of alcohol-treated larvae compared to the control group, which was alleviated in the livers of MgIG plus alcohol-treated larvae by whole-mount oil red O staining (Fig. 1a). The incidence of steatosis was significantly higher in the alcohol-treated group than in the control group (53% vs. 25%, *P* < 0.05) and was obviously reduced by MgIG pretreatment in a dose-dependent manner and NAC pretreatment (0.1 mg ml^−1^ MgIG + alcohol group: 26%, 0.05 mg ml^−1^ MgIG + alcohol group: 28%, 0.01 mg ml^−1^ MgIG + alcohol group: 46%, NAC + alcohol group: 30%) (Fig. 1b). We further confirmed diminished deposits of alcohol-induced fat droplets in MgIG plus alcohol-treated larvae by oil red O staining of frozen liver sections (Fig. 2a). We categorized “normal”, “medium”, or “severe” by varying degrees of lipid deposition in the livers of zebrafish by frozen liver sections (Fig. 2b). We found that nearly 20% of alcohol-treated larvae had “severe” lipid deposition in the liver by frozen liver sections, which was not found in other groups. The incidence of “normal” was lower in the alcohol-treated group than in the control group (36% vs. 73%), which was increased by MgIG pretreatment in a dose-dependent manner and NAC pretreatment (0.1 mg ml^−1^ MgIG + alcohol group: 70%, 0.05 mg ml^−1^ MgIG + alcohol group: 64%, 0.01 mg ml^−1^ MgIG + alcohol group: 40%, NAC + alcohol group: 56%) (Fig. 2c). Histological analysis further confirmed that microvesicular lipid droplets were present in the hepatocytes of alcohol-treated larvae, whereas no obvious fat droplets were found in the livers of MgIG plus alcohol-treated larvae and NAC plus alcohol-treated larvae (Fig. 1c). The levels of TC and TG increased in the alcohol-treated group. However, pretreatment of MgIG and NAC exhibited effects in terms of decreases in the level of both TC and TG (Fig. 1d, e).The results above strongly suggested that acute alcohol exposure induces hepatic steatosis in zebrafish and that MgIG has significant anti-steatosis activity.

### MgIG effectively attenuated acute alcohol-induced hepatomegaly in zebrafish larvae

**Fig. 3:**
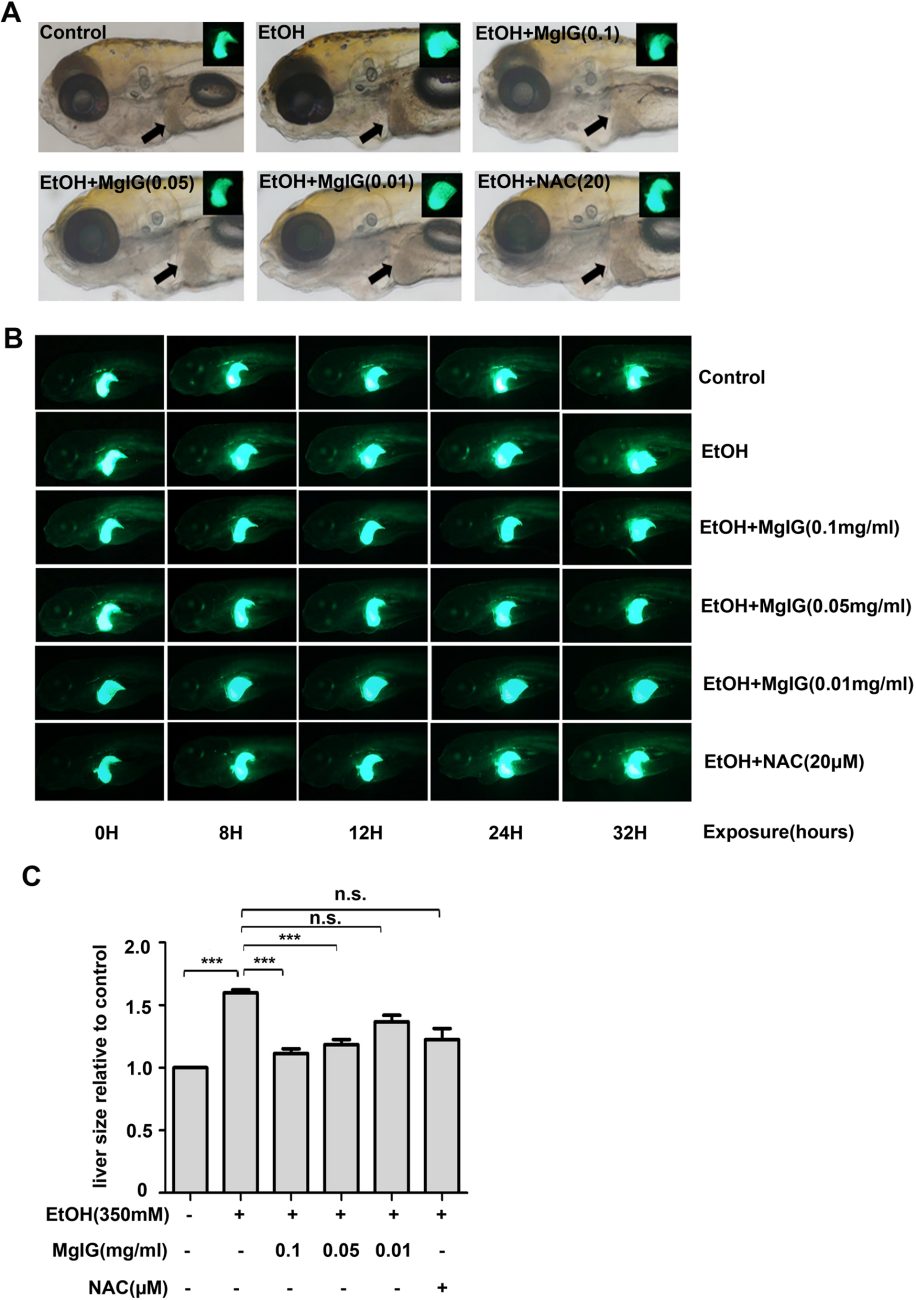
MgIG ameliorate acute alcohol induced-hepatomegaly in zebrafish Larvae. **a** Tg(*lfabp10a:eGFP*) zebrafish larvae were treated as indicated for 32 h and the typical pictures of each group were shown. Arrows point to the liver. Control group: 0% alcohol; EtOH group: 350 mM alcohol; EtOH + MgIG(0.1) group: 350 mM alcohol + 0.1 mg ml^−1^ MgIG; EtOH + MgIG(0.05) group: 350 mM alcohol + 0.05 mg ml^−1^ MgIG;EtOH + MgIG(0.01)group: 350 mM alcohol + 0.01 mg ml^−1^ MgIG; EtOH + NAC(20) group: 350 mM alcohol + 20 µM NAC. **b** Images of Tg(*lfabp10a:eGFP*) zebrafish larvae treated with 0% alcohol, 350 mM alcohol, pre-treated with 0.1 mg ml^−1^ MgIG, 0.05 mg ml^−1^ MgIG, 0.01 mg ml^−1^ MgIG, 20 µM NAC and co-exposed with 350 mM alcohol for 32 h. The morphological change of livers in zebrafish larvae were observed. Alcohol-treated larvae developed obvious hepatomegaly compared to the control group after 32 h of exposure, which was visibly ameliorated in MgIG-pretreated larvae. **c** The liver size was examined by image J software in zebrafish larvae exposed to 0% or 350 mM alcohol and larvae pre-treated with different concentrations of MgIG, 20 µM NAC and then co-exposed with 350 mM alcohol for 32 h. The liver size was normalized to control group. *P < 0.05, **P < 0.01, *n.s.*: no significant difference, by one-way ANOVA

Moreover, we observed that MgIG pretreatment could effectively improve hepatomegaly, especially at higher concentrations. We then traced the morphological changes in the livers of each group using liver-specific EGFP transgenic zebrafish. The results showed that alcohol-treated larvae developed obvious hepatomegaly compared to the control group after 32 h of exposure, which was visibly ameliorated in MgIG-pretreated larvae. Compared with higher concentrations, the hepatoprotective effect of 0.01 mg ml^−1^ MgIG was poor (Fig. 3a, b). As shown by the results of liver images, in comparison with the control group, the liver size in alcohol-treated group increased significantly, whereas liver size markedly decreased in the groups to which 0.1 mg ml^−1^ MgIG and 0.05 mg ml^−1^ MgIG had been administered. Although there was a decrease in liver size after 0.01 mg ml^−1^ MgIG and NAC pretreatment compared with the alcohol-treated group, the difference was not statistically significant (*P* > 0.05) (Fig. 3c).

### MgIG affected gene expression related to endoplasm reticulum (ER) stress in zebrafish larvae

**Fig. 4:**
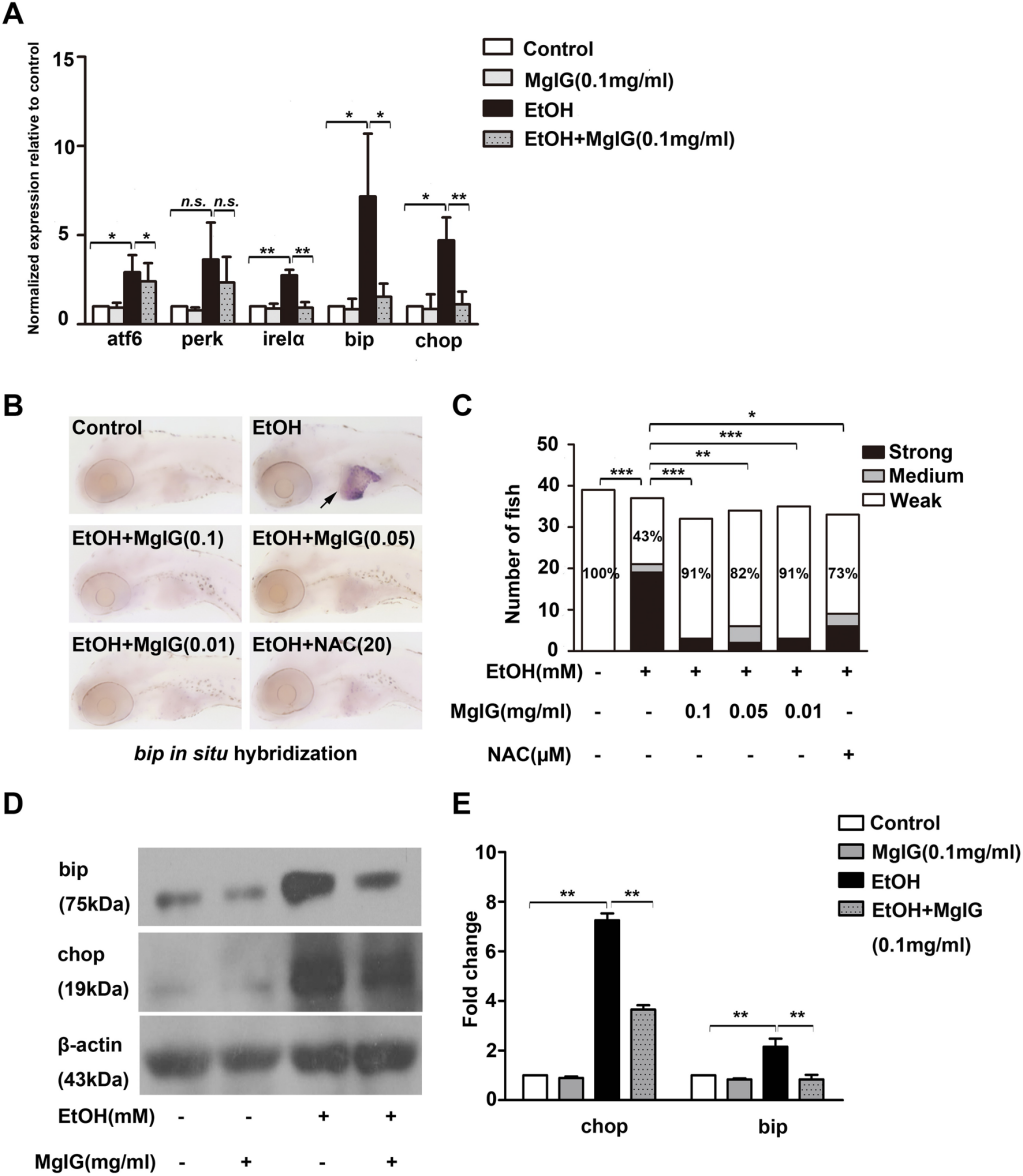
Effect of MgIG on alcohol-induced ER stress. **a** The relative *atf6, perk, irelα, bip* and *chop* mRNA expression was analyzed by qRT-PCR in the livers of larvae treated with 0% or 350 mM alcohol, 0.1 mg ml^−1^ MgIG, 350 mM alcohol + 0.1 mg ml^−1^ MgIG. *P < 0.05, **P < 0.01, *n.s.*: no significant difference, by one-way ANOVA. **b** The expression of *bip* was detected by whole-mount in situ hybridization. The typical pictures of each group were shown. **c** Quantification of *bip* expression is categorized “strong”, “medium”, or “weak”. Bar chart indicates the percentages of each category in each group, and the percentages of larvae in “weak” category is noted. *P < 0.05, **P < 0.01, ***P < 0.001, *n.s.*: no significant difference, by Chi-Square test. **d** and **e** Protein expression of bip and chop was examined by western blot in zebrafish larvae exposed to 0% or 350 mM alcohol, 0.1 mg ml^−1^ MgIG, or 350 mM alcohol + 0.1 mg ml^−1^ MgIG for 32 h, the degree of protein expression was normalized to β-actin. *P < 0.05, **P < 0.01, ***P < 0.001, by one-way ANOVA

Many studies have demonstrated that ER stress is involved in the development of hepatic steatosis and promotes the progression of ALD. We explored whether MgIG affected the function of ER to exert hepatoprotective effects in zebrafish larvae by qRT–PCR and whole-mount in situ hybridization. We found that the mRNA levels of genes involved in ER stress were consistently upregulated in the livers of alcohol-treated larvae compared to the control, including *atf6*, *ire1α*, *bip* and *chop* (*P* < 0.05). After MgIG pretreatment, the expression levels of these genes were remarkably reduced compared to the alcohol-treated group (*P* < 0.05) (Fig. 4a). Indeed, we observed that the percentage of “strong” expression of *bip* was significantly increased in the livers of alcohol-treated larvae compared to the control (*P* = 0.000), which was obviously reduced by MgIG pretreatment and NAC pretreatment (*P* < 0.05) (Fig. 4b, c and Additional file 2: Fig. S1). In addition, western blot analysis showed that the protein levels of bip, chop and perk were significantly increased in the alcohol-treated group compared to the control group and were downregulated after MgIG pretreatment (*P* < 0.01) (Fig. 4d, e and Additional file 2: Fig. S2). In summary, these data suggested that MgIG protects against acute alcoholic liver injury through improving ER stress.

### MgIG affected the expression of genes associated with lipid metabolism pathways in zebrafish larvae

**Fig. 5:**
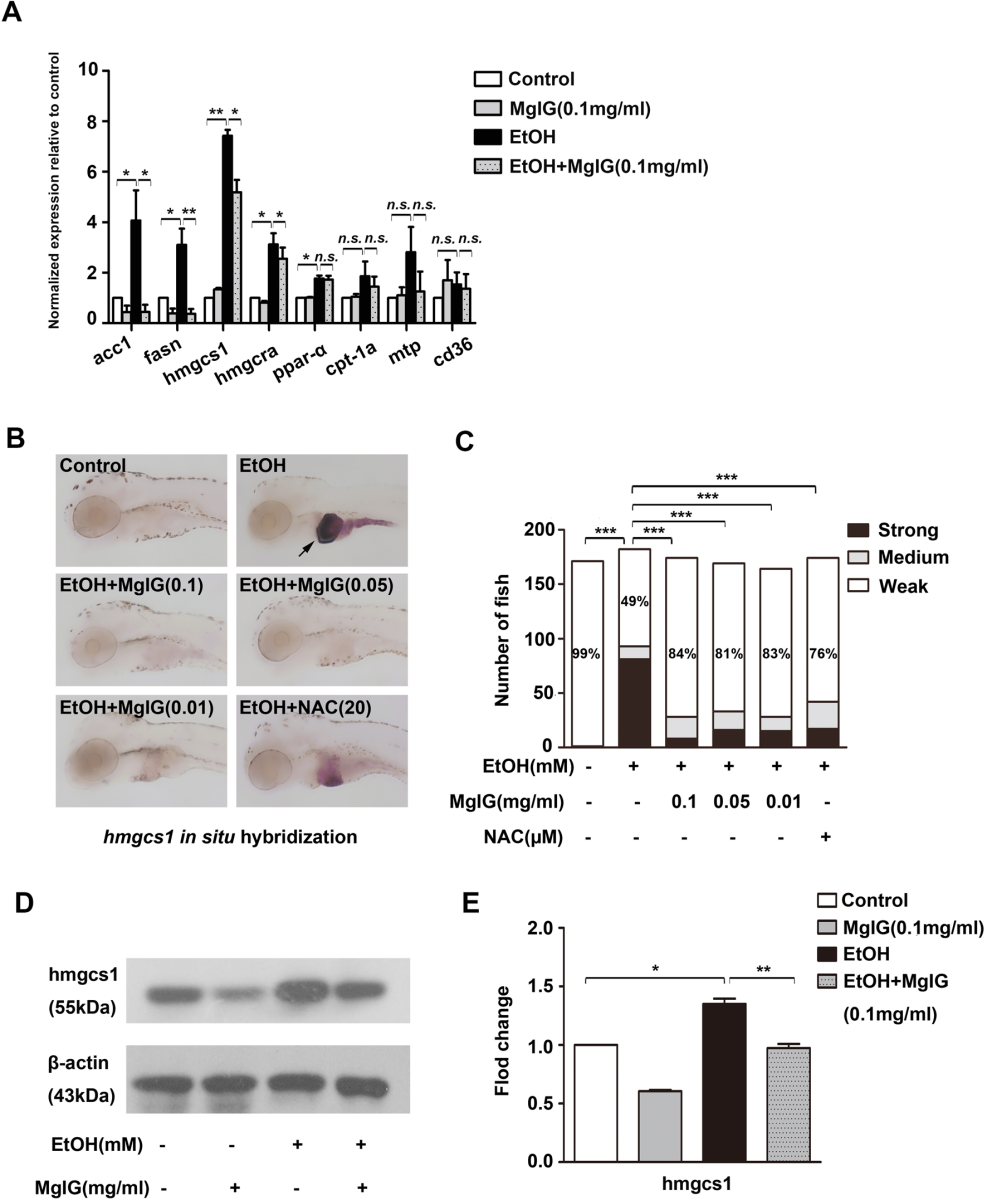
Effect of MgIG on alcohol-induced lipid metabolism dysfunction. **a** The relative *acc1, fasn, hmgcs1*, *hmgcra, ppar-α, cpt-1*, *mtp, cd36* mRNA expression was analyzed by qRT-PCR in the livers of larvae treated with 0% or 350 mM alcohol, 0.1 mg ml^−1^ MgIG, or 350 mM alcohol + 0.1 mg ml^−1^ MgIG. *P < 0.05, **P < 0.01, ***P < 0.001, *n.s.*: no significant difference, by one-way ANOVA. **b** The expression of *hmgcs1* was detected by whole-mount in situ hybridization. The typical pictures of each group were shown. **c** Quantification of *hmgcs1* expression is categorized “strong”, “medium”, or “weak”. Bar chart indicates the percentages of each category in each group, and the percentages of larvae in “weak” category is noted. *P < 0.05, **P < 0.01, ***P < 0.001, *n.s.*: no significant difference, by Chi-Square test. **d** and **e** Protein expression of hmgcs1 was examined by western blot in larvae exposed to 0% or 350 mM alcohol, 0.1 mg ml^−1^ MgIG, or 350 mM alcohol + 0.1 mg ml^−1^ MgIG for 32 h, the degree of protein expression was normalized to β-actin. *P < 0.05, **P < 0.01, ***P < 0.001, by one-way ANOVA

To investigate the mechanism by which MgIG reduced hepatic lipid accumulation, genes involved in lipid metabolism were examined by qRT-PCR and whole-mount in situ hybridization. The levels of key lipogenic enzymes related to fatty acid synthesis cholesterol synthesis were significantly increased in the alcohol-treated group compared to the control group, including *acc1*, *fasn*, *hmgcs1*, *and hmgcra,* which were reduced after MgIG pretreatment (*P* < 0.05). Unexpectedly, we found that the expression levels of *ppar-*α were significantly higher in the alcohol-treated group than in the control group (*P* < 0.05). MgIG pretreatment had no effect on the expression levels of *ppar-α*, *cpt-1a*, *mtp* and *cd36* (*P* > 0.05) (Fig. 5a). Indeed, we found that the percentage of *hmgcs1* with “strong” expression was significantly increased in the livers of alcohol-treated larvae compared to the control (*P* = 0.000), which was obviously reduced by MgIG pretreatment and NAC pretreatment (*P* = 0.000) (Fig. 5b, c and Additional file 2: Fig. S3). In addition, western blot analysis showed that the protein levels of hmgcs1 were significantly increased in the alcohol-treated group compared to the control group and were downregulated after MgIG pretreatment (*P* < 0.05) (Fig. 5d, e). Taken together, these results indicated that MgIG reduces hepatic lipid accumulation in zebrafish larvae, probably by ameliorating the disorder of lipid metabolism.

## Discussion

Alcohol abuse responds to the global burden of liver injury and is one of the leading causes of morbidity and mortality. According to the WHO, it is the cause of 4.5% of diseases in the world and 4% of deaths worldwide [31, 32]. Although most body organs are affected by acute alcohol intoxication and chronic alcohol use, severe alcohol-induced diseases are most notable in the liver because it is the primary site of alcohol metabolism. Hepatic steatosis is the earliest response to heavy drinking and is characterized by the deposition of excessive lipids in hepatocytes. Steatotic hepatocytes lose the ability to detoxify and become more sensitive to toxins and can progress to steatohepatitis, liver fibrosis, cirrhosis and even hepatocellular carcinoma [33]. Although a considerable amount of attention has been devoted to acute alcohol-induced liver injury, the underlying mechanisms are unclear, and therapeutic drugs are currently lacking.

Magnesium isoglycyrrhizinate (MgIG) is extracted from the roots of *Glycyrrhiza glabra* (licorice). It is widely used in the treatment of various liver diseases due to its well-recognized transaminase-lowering effect in clinical applications. The mechanisms involved in MgIG’s hepatoprotective effects mainly include its antioxidative, anti-inflammatory, energy homeostasis regulation and antiapoptotic activities [34–36]. Studies have indicated that MgIG inhibits the inflammatory response by blocking the STAT3 pathway in a partial hepatectomy model [37]. MgIG also showed protective effects on nonalcoholic liver diseases [38], immune-mediated liver injury [39] and drug-induced liver injury [40, 41]. A study from Guiqiang Wang’s group demonstrated that MgIG retards the development of alcohol-induced liver injury by regulating neutrophil activity and hepatic oxidative stress using a chronic plus binge alcohol feeding mouse model [42]. A recent report showed that MgIG could reduce lipid deposition induced by acute alcohol consumption through blockade of the Hedgehog pathway in vitro [43]. However, data on the effects of MgIG on acute alcohol exposure-induced hepatic steatosis in vivo are very limited. Given the strong effects of MgIG, we hypothesized that this compound might exhibit therapeutic properties in the context of acute alcohol-induced hepatic steatosis.

Zebrafish models of liver disease have been well established for years and are useful for screening effective agents in vivo and studying molecular pathogenesis [29, 44]. Passseri et al. first described a novel zebrafish model for acute alcohol-induced hepatic steatosis and had been applied to some drug studies [6, 19]. In the current study, we utilized this model to investigate the hepatoprotective effects and mechanism underlying the effect of MgIG on acute alcohol-induced hepatic steatosis. Oil red O staining and histological examination showed a reduced incidence of hepatic steatosis and less lipid droplet accumulation in the livers of MgIG-pretreated zebrafish than in the livers of alcohol-treated zebrafish. Moreover, MgIG significantly decreased the levels of TC and TG in the livers of zebrafish larvae. We also found that MgIG improved hepatomegaly using liver-specific EGFP transgenic zebrafish. The protective effects of 0.1 mg ml^−1^ MgIG and 0.05 mg ml^−1^ MgIG were superior to those of 0.01 mg ml^−1^ MgIG. Taken together, these data provide evidence that MgIG pretreatment could effectively attenuate acute alcohol-induced hepatic steatosis in a zebrafish model.

Alcohol metabolism-generated aldehydes are highly reactive and can lead to protein adduct accumulation, thereby inducing ER stress [4, 8]. The unfolded protein response transducers Atf6, Ire-1α and Perk were all activated upon alcohol exposure. The three UPR pathways are regulated by Bip, a protein chaperone that binds and releases mediators of each pathway. As a consequence, the transcription factor Chop, which is known to modulate cell death, was upregulated. Numerous studies have shown that ER stress contributes to alcohol-induced steatosis [45, 46]. A previous study reported that glycyrrhizin can repress total parenteral nutrition-associated acute liver injury in rats by suppressing ER stress [47]. Another report showed that glycyrrhizic acid (GA) had highly significant ROS quenching activity, thereby blocking the activation of ER stress and the MAPK pathway in UV-B-irradiated human skin fibroblasts [48]. In the present study, we found that MgIG markedly reduced the expression of genes and proteins associated with ER stress in zebrafish liver, indicating that MgIG may improve ER stress to protect liver cells.

Regarding the molecular mechanism by which MgIG ameliorates hepatic lipid accumulation, we then detected the expression of genes associated with lipid metabolism. Previous studies have shown that MgIG can modulate lipid metabolism in rats [49] and hepatocytes [50]. In the present study, our results showed that the mRNA levels of a*cc1*, *fasn*, *hmgcs1*, and *hmgcra* were all significantly increased in the livers of zebrafish in the alcohol-treated group and were suppressed by MgIG pretreatment. Unexpectedly, the mRNA levels of *ppar-*α were also significantly increased in the livers of zebrafish in the alcohol-treated group, and MgIG pretreatment had no impact on the expression of *ppar-α*, *cpt1a*, *mtp* and *cd36*. These data suggested that the protective effects of MgIG might not be related to fatty acid oxidation, lipid binding and transport pathways. The protective effects of MgIG occur through regulating the lipogenic pathway, which results in the prevention of lipid accumulation in the liver in a zebrafish model.

In summary, the current study demonstrated that MgIG could alleviate acute alcohol-induced hepatic steatosis in zebrafish larvae. MgIG pretreatment significantly suppressed acute alcohol-induced ER stress and lipid metabolism dysfunction. These factors may work synergistically and contribute to the protection of MgIG against acute alcohol-induced steatosis. Although our data showed that MgIG may have an effect on ER stress and lipid metabolism, more studies are needed to research the precise molecular mechanism by which MgIG exerts hepatoprotective effects in ALD.

## Conclusions

This study developed and validated a larval zebrafish hepatic steatosis model induced by alcohol that could be used for the efficacy assessment of anti-steatosis drugs in vivo. We demonstrated that MgIG might play a hepatoprotective role in acute alcohol-induced steatosis in zebrafish larvae by improving ER stress and inhibiting lipogenesis.

## Supplementary Information

**Additional file 1: Table S1**. Primer sequences used for quantitative RT-PCR.

**Additional file 2. Fig S1**. Representative images of bip expression in the livers of zebrafish by whole-mount in situ hybridization. **Fig S2**. MgIG had an effect on the expression level of Perk protein by western-blot. **Fig S3**. Representative images of hmgcs1 expression in the livers of zebrafish by whole-mount in situ hybridization.

## Data Availability

All date generated or analyzed during this study are included in this article.

## Abbreviations

ALD: Alcoholic liver disease
MgIG: Magnesium isoglycyrrhizinate
ER stress: Endoplasmic reticulum stress
dpf: Days post fertilization
EtOH: Ethanol
NAC: *N*-Acetylcysteine
H&E: Hematoxylin and eosin
acc1: Acetyl-CoA carboxylase
fasn: Fatty acid synthase
hmgcs1: 3-Hydroxy-3-methylglutary-CoA synthase 1 (soluble)
hmgcra: 3-Hydroxy-3-methylglutary-CoA reductase a
ppar-α: Peroxisome proliferator-activated receptor α
cpt-1a: Carnitine palmitoyltransferase 1a
mtp: Microsomal triglyceride transfer protein
cd36: Fatty acid translocase
atf6: Activating transcription factor 6
perk: RNA-dependent protein kinase(PKR)-like ER kinase
irelα: Inositol-requiring enzyme 1a
bip: Binding immunoglobulin protein
chop: CCAAT/enhancer-binding protein homologous protein
