# Supplementary material for: Magnesium isoglycyrrhizinate attenuates acute alcohol-induced hepatic steatosis in a zebrafish model by regulating lipid metabolism and ER stress
Source: Nutr Metab (Lond). 2022 Mar 24;19:23. doi: 10.1186/s12986-022-00655-7 (PMC8944020; doi:10.1186/s12986-022-00655-7)
Supplement: Supplementary file 1 — Additional file 1: Table S1. Primer sequences used for quantitative RT-PCR. [file 12986_2022_655_MOESM1_ESM.docx]

**Additional file 1: Table S1. Primer sequences used for quantitative RT-PCR**

| Gene | Forward Primer | Reverse Primer |
| --- | --- | --- |
| *acc1* | GCAAGTGTGGTTCCCTGATT | TCATGAAGGTCAGCGAACTG |
| *fasn* | GAGAAAGCTTGCCAAACAGG | GAGGGTCTTGCAGGAGACAG |
| *hmgcs1* | CTCACTCGTGTGGACGAGAA | GATACGGGGCATCTTCTTGA |
| *hmgcra* | CTGAGGCTCTGGTGGACGTG | GCAGCTACGATGTTGGCG |
| *ppar-*α | CGTCGTCAGGTGTTTACGGT | AGGCACTTCTGGAATCGACA |
| *cpt-1a* | ACTCTCGATGGACCCTGTGA | CTGGATGAAGGCATCTGGAC |
| *atf6* | CTGTGGTGAAACCTCCACCT | CATGGTGACCACAGGAGATG |
| *perk* | TGGGCTCTGAAGAGTTCGAT | TGTGAGCCTTCTCCGTCTTT |
| *ire1*α | TGACGTGGTGGAAGTTGGTA | ACGGATCACATTGGGATGTT |
| *bip* | AAGAGGCCGAAGAGAAGGAC | AGCAGCAGAGCCTCGAAATA |
| *chop* | AAGGAAAGTGCAGGAGCTGA | TCACGCTCTCCACAAGAAGA |
| *mtp* | CTCAGCTGGTGGATGCAGTA | ATCTCTGTGCTGCCGATCTT |
| *cd36* | AGGCCACTGTGAACCTGAAG | AAGTTGGGGTTCATTCCGAC |
| *eef1a1* | TACTTCTCAGGCTGACTGTG | ATCTTCTTGATGTATGCGCT |
