# Supplementary material for: Magnesium isoglycyrrhizinate attenuates acute alcohol-induced hepatic steatosis in a zebrafish model by regulating lipid metabolism and ER stress
Source: Nutr Metab (Lond). 2022 Mar 24;19:23. doi: 10.1186/s12986-022-00655-7 (PMC8944020; doi:10.1186/s12986-022-00655-7)
Supplement: Supplementary file 2 — Additional file 2. Fig S1. Representative images of bip expression in the livers of zebrafish by whole-mount in situ hybridization. Fig S2. MgIG had an effect on the expression level of Perk protein by western-blot. Fig S3. Representative images of hmgcs1 expression in the livers of zebrafish by whole-mount in situ hybridization. [file 12986_2022_655_MOESM2_ESM.docx]

**Additional file 2**

**
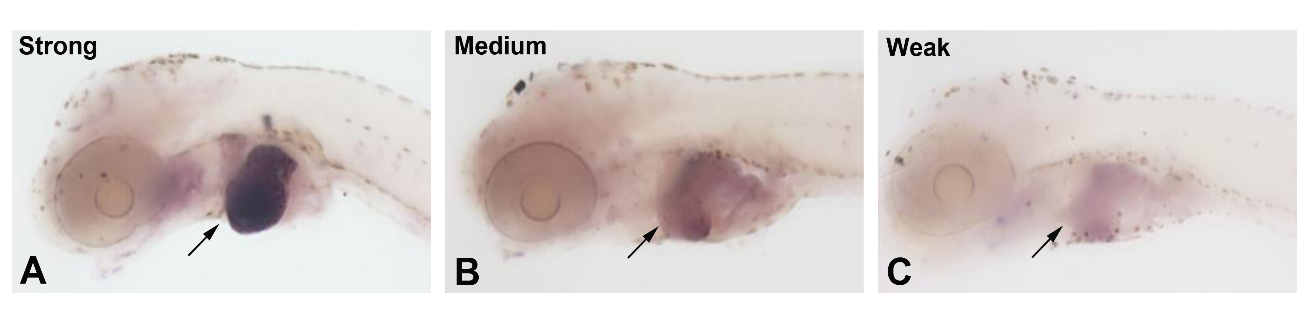
**

**Fig. S1** Zebrafish Larvae of 5 dpf treated with 0% alcohol, 350mM alcohol，pre-treated with 0.1 mg ml^-1^ MgIG、0.05 mg ml^-1^ MgIG、0.01 mg ml^-1^ MgIG 、20μM NAC and co-exposed with 350mM alcohol for 32 hours. We categorized “Strong”, “Medium”, or “Weak” by varying degrees of *bip* expression in the livers of zebrafish by whole-mount *in situ* hybridization. Representative images of “Strong”, “Medium”, or “Weak” were shown. Black arrow indicated the liver (×63 magnification).


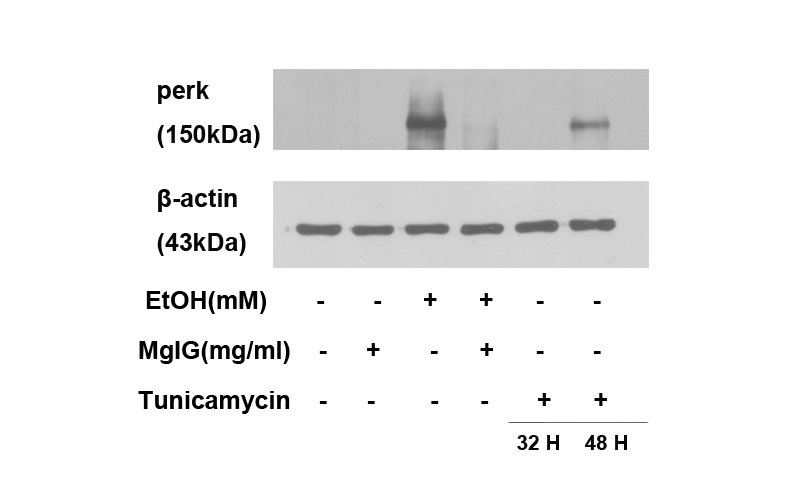


**Fig. S2** Zebrafish Larvae of 5 dpf treated with 0% alcohol, 0.1 mg ml^-1^ MgIG， 350mM alcohol or pre-treated with 0.1 mg ml^-1^ MgIG and co-exposed with 350mM alcohol for 32 hours. Tunicamycin (1 μM) as a ER stress inducer was given to the larvae at 5dpf for 32 hours or 48 hours. Protein expression of perk was examined by western blot in zebrafish larvae, the degree of protein expression was normalized to β-actin.

**
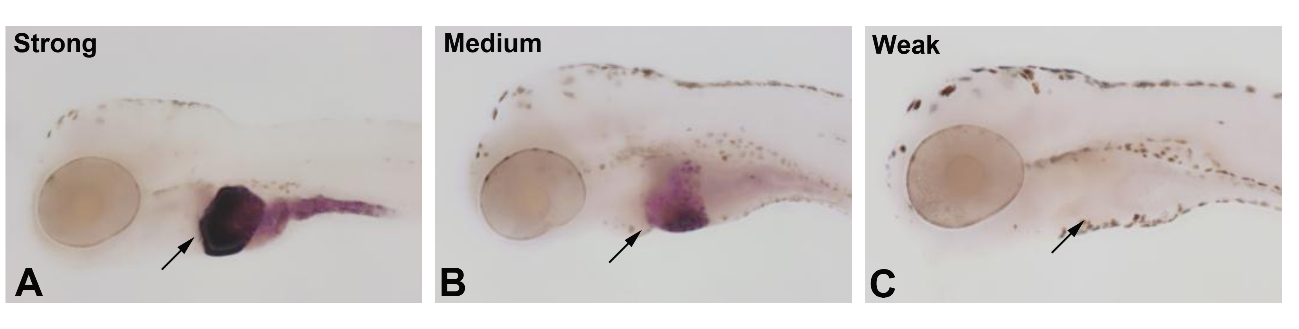
**

**Fig. S3** Zebrafish Larvae of 5 dpf treated with 0% alcohol, 350mM alcohol，pre-treated with 0.1 mg ml^-1^ MgIG、0.05 mg ml^-1^ MgIG、0.01 mg ml^-1^ MgIG 、20μM NAC and co-exposed with 350mM alcohol for 32 hours. We categorized “Strong”, “Medium”, or “Weak” by varying degrees of *hmgcs1* expression in the livers of zebrafish by whole-mount *in situ* hybridization. Representative images of “Strong”, “Medium”, or “Weak” were shown. Black arrow indicated the liver (×63 magnification).
